# The St. John Ambulance Association and the Voluntary Aid Detachments

**Published:** 1910-12-10

**Authors:** 


### The Royal Army Medical Corps Section.

### THE ST. JOHN AMBULANCE ASSOCIATION AND THE VOLUNTARY AID DETACHMENTS.

In our issue of August 6, when commenting on the appointment of a War Office Advisory Committee for the Voluntary Aid Detachments, we expressed approval of the step taken, as it might materially assist in removing some of the difficulties that had arisen in the formation of these detachments. Of these difficulties the most important, and the one that militated most against their success, was the somewhat strained relations that had developed between the St. John Ambulance Association and the British Red Cross Society. Into the causes that led to this unfortunate state of matters it is unnecessary to enter, but we are glad to learn that as the outcome of negotiations that have taken place, and on which no doubt the influence of the respective members of the Advisory Council was brought to bear, a War Office letter has recently been circulated to County Associations in England and Wales that seems to us calculated to remove a very disturbing element that has been at work. To understand this it must be remembered that the St. John Association organised what were known as St. John Ambulance County Companies for aid to the sick and wounded of the Territorial Force in the event of invasion. In our August article we said we could not approve of this action, as the St. John Association thus put itself into competition with the British Red Cross Society in a matter specially handed over to the latter by the military authorities. The War Office letter just issued deals specifically with this point, and lays down clearly the position of the St. John Ambulance Association in connection with the scheme for the organisation of voluntary aid in England and Wales. It makes it clear that in all the counties the Red Cross County Committees are to have the refusal of the task of raising the Voluntary Aid Detachments required, and that it is only in the case of their inability from local or other circumstances to carry out the undertaking that the Territorial Force Association of the county is to call on the St. John Ambulance Association to do the work. When this happens, the Army Council are prepared to accept "Brigade" and "County" Companies of the St. John Ambulance Association so raised, and to regard them as the equivalent of the Voluntary Aid Detachments of the Red Cross Society. To this arrangement the St. John has given its agreement, and it has also undertaken, as heretofore, to give its valuable assistance to the Red Cross County Committees by holding classes in first aid for the Voluntary Aid Detachments, so that the members of these detachments may obtain the necessary qualifying certificates. We think the result arrived at is in every way creditable to the St. John Ambulance Association, and is the proper solution of what was rapidly becoming a very undesirable and impossible position of matters. To see two bodies competing with each other in somewhat of a hostile spirit for the carrying on of a popular and patriotic movement could not but be regarded as unfortunate, and even unseemly. This has now been avoided, and it is a matter for congratulation. We are also glad that there has, at the same time, been established the very proper principle that recognition should be given to those other bodies, besides the St. John Association, who grant certificates of first aid of undoubted excellence. It would have been unfair that one particular certificate should be demanded from candidates for the detachments to the exclusion of all others, especially if those desirous to join were already possessed of another equally good for the purpose in hand, as many of them were. To make one certificate privileged, and to disallow all others, could only hinder recruiting and hamper the progress of the movement. What is needed in work of this kind is to give people every facility for joining it, and not to place any obstacles in the way. The War Office letter points out that the St. John Association is available for teaching purposes, but very properly does not insist on its selection to the exclusion of other bodies. It is gratifying to know that forty-seven Territorial County Associations in England and Wales, and all of those in Scotland, have asked the Red Cross County Committees to raise the detachments. That the scheme is popular is shown by the fact that up to October 20 of this year there have been raised by the British Red Cross Society, and registered and numbered at the War Office, no fewer than two hundred and two detachments, of whom forty-four are male and one hundred and fifty-three female. These represent a *personnel* of 1,871 men and 4,351 women, or a combined total of 6,222 for England, Wales, and Scotland. Of the different counties Gloucester holds the lead with forty-five detachments, the next to it being Devon with twenty-three. After the latter come the county of the City of London with twenty-one, Essex with eighteen, and Hampshire with fourteen. As to the practicability of the scheme we are hopeful, as we are aware that several very encouraging demonstrations have been given by different Voluntary Aid Detachments, and notably by those in the vicinity of Oakham. These latter were tested by being called upon to deal with fifty supposed wounded, who were labelled with definite injuries and were distributed along the roads and lanes in a particular district. In accordance with instructions issued to them, the detachments had to render first aid, to transport the wounded by improvised stretchers or other means to a specially selected field, where field kitchens had been made and the necessary medical comforts got ready. When there, short records of the cases were made, the dressings readjusted, the temperatures taken, and within two hours arrangements completed for the disposal under shelter during the night of all the fifty casualties. Very great assistance was rendered by the boy and girl scouts in the neighbourhood, who were invaluable in procuring from the adjoining houses and their occupants the loan of many necessary articles for the feeding and comfort of those under treatment, with the result that the whole demonstration was carried out not only without cost but in a regular and methodical manner, thus furnishing a striking contrast to the confusion and excitement that would have undoubtedly characterised the work had it not been in the hands of trained and disciplined workers. Such practical training is the best preparation for the realities of war, and the excellent way it was carried through is a happy augury of the advantages it would confer on our wounded Territorial soldiers should the occasion arise.

